# The implication of *BRAF* mutation in advanced colorectal cancer

**DOI:** 10.1007/s11845-021-02689-x

**Published:** 2021-12-08

**Authors:** Emma O’Riordan, Michael William Bennett, Louise Daly, Derek G Power

**Affiliations:** 1 School of Medicine, University College Cork, Cork, Republic of Ireland; 2 Department of Histopathology, Cork University Hospital, Cork, Republic of Ireland; 3 School of Food & Nutritional Sciences, University College Cork, Cork, Republic of Ireland; 4 Department of Medical Oncology, Mercy & Cork University Hospitals, Cork, Republic of Ireland

**Keywords:** *BRAF*, Cancer, Colorectal, Mutation

## Abstract

**Background:**

Advanced colorectal cancer (CRC) is frequently a lethal disease. Mutations in the *BRAF* gene is a key driver in CRC pathogenesis and confers a poor prognosis. To date, Irish data on this molecular subtype of CRC is lacking.

**Aims:**

Our aim was to compare the natural history of Irish patients with *BRAF* (*BRAF*^MUT^) metastatic CRC with a control group of metastatic CRC patients without *BRAF* mutation (*BRAF*^WT^ wild- type).

**Method:**

A retrospective observational analysis of advanced CRC patients with known *BRAF*^MUT^ was conducted by chart review. *BRAF*^MUT^ patients were identified from the Cork University Hospital (CUH) histopathology database. Controls with known *BRAF*^WT^ were randomly selected from the database. Demographic characteristics and clinicopathological data were recorded. Survival was assessed with Kaplan–Meier curve/Cox proportional hazard models.

**Results:**

Twenty patients with *BRAF*^MUT^ and 36 with *BRAF*^WT^ were studied. *BRAF*^MUT^ were more likely female (75% vs 33%, *p* = 0.007) and right-sided (65% vs 31.4%, *p* = 0.033). Median overall survival was lower in *BRAF*^MUT^ group (17.3 months (95% CI 0–40.8)) compared to patients with *BRAF*^WT^ (median survival not reached, log rank *p* = 0.001). On multivariate analysis, *BRAF*^MUT^ was independently associated with an increased risk of mortality (HR 12.76 (95% CI 3.15–51.7), *p* < 0.001).

**Conclusion:**

*BRAF*^MUT^ advanced colorectal cancer was associated with significantly reduced overall survival in this Irish CRC population. Knowledge of mutation status should now be considered standard of care and should dictate management. Surgeons should be aware of this genetic signature as the natural history of the disease may mitigate against an aggressive surgical strategy. A prospective study should be conducted to further corroborate these findings.

## Introduction

Colorectal cancer (CRC) is the second most diagnosed malignancy in Ireland, with an annual incidence of 2767 new cases [1]. It accounts for 13% of cancer deaths in males and 10% in females or an average of 1010 deaths per year [2]. This represents a major burden on the Irish Health Service. Colorectal cancer has a complex pathogenesis involving both genetic and environmental factors [3]. There are several defined genetic pathways along which sporadic CRC can develop, characterised by distinctive models of genetic and epigenetic instability [4, 5]. These include chromosomal instability (CIN), DNA mismatch repair insufficiency (MMR), and CpG Island Methylator status (CIMP) [6, 7]. Alterations in the latter include *BRAF* mutations, and can result in distinct pathologic and clinical characteristics.

Emerging evidence suggests that the *BRAF* mutation may be a marker of poor prognosis in CRC patients [8–10]. *BRAF* gene encodes for a signal transduction protein found downstream of *KRAS* in the Mitogen activated protein kinase (MAPK) pathway [11, 12]. *BRAF* is involved in regulation of the (*MAPK*)/*ERK* signalling pathway which plays a role in cell proliferation, differentiation, migration, and apoptosis [13, 14]. Mutations in *BRAF* result in over-expression of this pathway, leading to uncontrolled cell proliferation [15]. Recent studies have estimated that approximately 8–15% of CRC patients carry a *BRAF* mutation [16]. Various international trials have also found *BRAF* mutations in CRC present early in disease progression (stage I/II) and occur mainly in right-sided tumours, females, and those over the age of fifty at diagnosis [17, 18].

Many studies have correlated the presence of a *BRAF* mutation in advanced CRC with metastatic disease, poor outcomes following oncological treatment, and reduced overall survival when compared with *BRAF*^WT^ (wild-type) disease [8–10]. The recent BEACON trial illustrates how *BRAF*^MUT^ CRC is relatively chemo-insensitive and responds poorly to conventional chemotherapy regimes [16]. It has also been shown that *BRAF*^MUT^ tumours fail to respond to epidermal growth factor (EGFR) inhibitors [16, 19]. Therefore, it has been suggested that knowledge of the *BRAF* mutation’s status in CRC may guide treatment options, and may mitigate against an aggressive surgical or oncological strategy.

Whilst *BRAF*^*MUT*^ CRC has been increasingly studied internationally, there remains a paucity of data to define how *BRAF*^MUT^ affects the Irish CRC population, and its incidence and impact remain unknown. It has been reported that the incidence of *BRAF* mutations in other diseases differs between distinct populations. A 2015 Irish study by van den Hurk et al., which examined the incidence of *BRAF*^MUT^ melanoma in both Ireland and internationally, found *BRAF*^MUT^ melanoma rates differed significantly between Irish patients (19%) and their Belgian counterparts (43%) [20]. The lack of clinical information regarding the impact of *BRAF*^MUT^ CRC in an Irish population remains a key gap in the scientific knowledge basis, which needs urgent clarification in order to optimise oncological and surgical treatment for Irish CRC patients.

The specific aims of this study were to assess the clinicopathological characteristics of a cohort of Irish patients with *BRAF*^MUT^ metastatic colorectal cancer, and to compare this with a matched control group with *BRAF*^WT^, and to examine differences in overall survival (OS) between the two groups.

## Methods

Ethical approval for this study was sought from the Clinical Research Ethics Committee of the Cork Teaching Hospitals (CREC) in February 2018. Ethical approval was granted in December 2018.

This study was a retrospective observational analysis carried out by means of chart review, and was conducted between the Mercy University Hospital (MUH) and Cork University Hospital (CUH). Charts were obtained by consulting the CUH pathology database and extracting all patient medical record numbers (MRNs) for whom *BRAF* testing had been conducted. *BRAF* testing was conducted by single-gene PCR, and all *BRAF*^MUT^ patients had the V600E subtype. All patients with *BRAF*^MUT^ disease were taken to form the study cohort, and a randomly selected control group of *BRAF*^WT^ patients was formed using SPSS v.25.

Table: Inclusion criteria

**Table:** 

Inclusion criteria	Exclusion criteria
> 18 years of age	< 18 years of age
Diagnosis of metastatic CRC	*BRAF* status unknown
*BRAF* status known	
Received systemic chemotherapy	

A total of n = 416 patients with histologically or radiologically confirmed metastatic CRC were tested for *BRAF* status in Cork University Hospital (CUH) and The Mercy University Hospital (MUH) during the study period (Jan 2014 to December 2018). The standard molecular profile performed in all patients with metastatic CRC includes *KRAS*, *NRAS*, *BRAF*, and *MMR*.

Twenty of these patients tested positive for *BRAF*^MUT^ CRC, and these patients formed the study cohort. A randomised sample group of 40 patients was selected from those who tested negative for the mutation (*BRAF*^WT^ group) to form the 2:1 control group, using SPSS v.25. Four patients were excluded from the control group as they did not meet inclusion criteria for the study.

Figure: Flowchart of patient inclusion

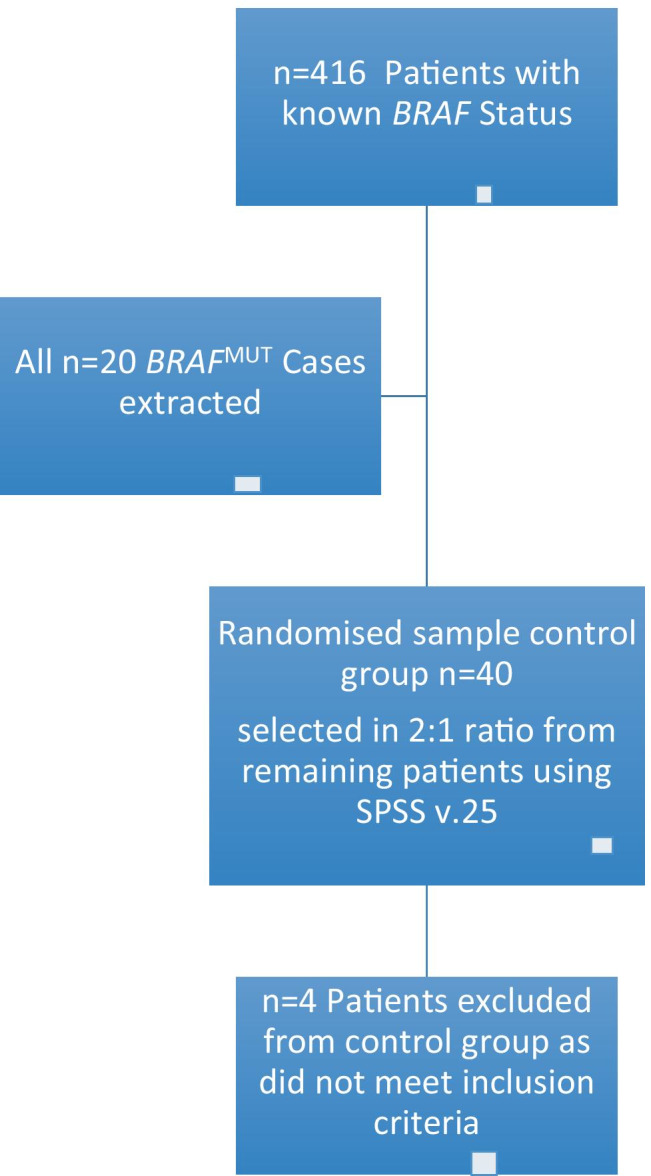



The retrospective observational analysis was carried out by means of chart review. Each chart was analysed on an individual basis with information recorded in a Microsoft Excel spreadsheet. The spreadsheet was stored on a password protected laptop in an encrypted file folder, with each patient designated an individual number to maintain anonymity, in compliance with GDPR.

A data collection sheet was designed and data collected in accordance with this. Data obtained from the patient’s charts included (1) demographic details, such as age and gender at diagnosis; (2) clinicopathological data, including tumour location, stage, and metastasis at diagnosis; and (3) oncological outcomes including date of diagnosis and date of death, in order to record overall survivals.

SPSS version 25 (SPSS, Inc., Chicago, Illinois) was used for statistical analysis. Population demographics and chemotherapy data were assessed using a t-test, Mann–Whitney U test, or chi-square test as appropriate. Overall survival (OS) was assessed using Kaplan–Meier curve and Cox proportional hazard models. Results were considered statistically significant when *p* < 0.05.

## Results

**Fig. 1:**
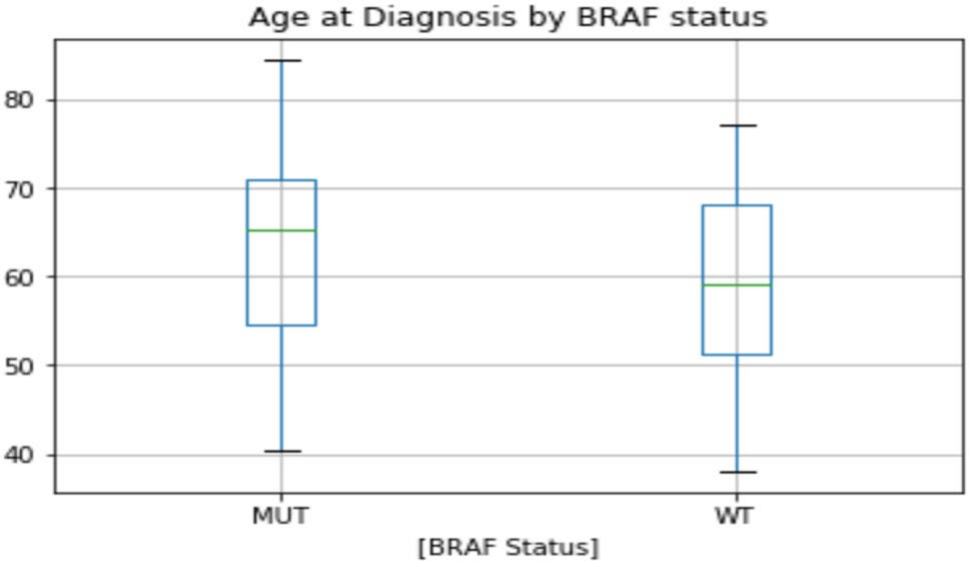
Age at diagnosis (median, range and interquartile range)

A total of 56 patients were included in this study. The study group consisted of 20 patients with *BRAF*^MUT^ advanced colorectal cancer, while 36 patients who had advanced colorectal cancer but who did not have the genetic mutation (*BRAF* wild-type, *BRAF*^WT^) were included as controls. There was no difference in age between groups with a median age of 65 years in the *BRAF*^MUT^ group and 59 years in the *BRAF*^WT^ group (*p* = n.s.; Fig. 1).

**Fig. 2:**
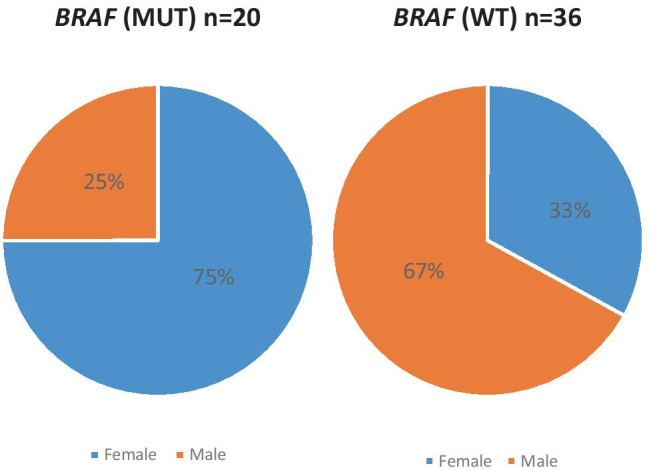
Gender in patients with *BRAF*-Mutation and *BRAF*- wild type

However, patients with the *BRAF* mutation were far more likely to be of female gender (75%) than those with *BRAF* wild-type (33%, *p* < 0.007; Fig. 2).

The characteristics of the tumours are seen in Table 1. Overall, 53.6% of the tumours had evidence of metastatic disease at the time of their original diagnosis, but there was no significant difference noted between the two groups. There was also no difference in histological type between groups, but *BRAF*^MUT^ tumours were more likely to be poorly differentiated when compared with the *BRAF*^WT^ subgroup (45% vs 13.9%, *p* = 0.038). A total of 81% (29 patients) of the *BRAF*^WT^ cases harboured a *KRAS* mutation, whereas none of the *BRAF*^MUT^ tumours did so (p < 0.0001).

**Table 1:** Tumour characteristics at diagnosis

	*BRAF*-mutated (*n* = 20)	*BRAF*-wild type (*n* = 36)	*p*
Tumour stage (At Diagnosis)			*p* = 0.26
Stage 1	0	1 (2.78%)	
Stage 2	1 (5%)	5 (13.9%)	
Stage 3	9 (45%)	8 (22.2%)	
Stage 4	10 (50%)	22 (61.1%)	
Histological type			*p* = 1.0
Adenocarcinoma	19 (95%)	36 (100%)	
Mucinous	1 (5%)	0	
Mutation			
MMR	3 (15%)	4 (11.1%)	*p* = 1.0
KRAS	0	29 (80.5%)	*p* < .00001
Poorly differentiated	9 (45%)	5 (13.9%)	

**Fig. 3:**
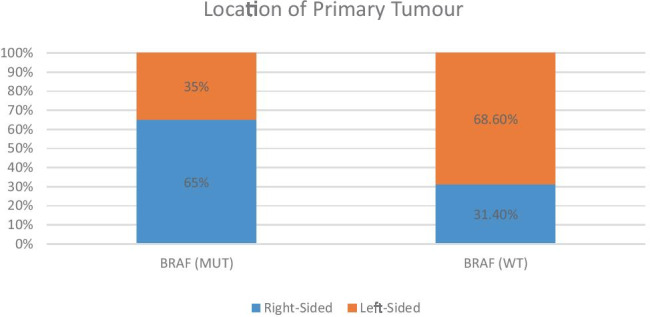
Location of primary tumour

*BRAF*-mutated tumours were more likely to be right-sided (65%) than *BRAF* wild-type tumours (31%, *p* = 0.033; Fig. 3).

Table 2 shows the treatments offered to patients. All but 2 patients (one in each of *BRAF*^MUT^ and *BRAF*^WT^ groups) were treated with chemotherapy, and an average of 3 lines of treatment were administered. There were no differences in chemotherapy regimens between groups.

**Table 2:** Treatments

	*BRAF*-Mutated (*n* = 20)	*BRAF*-Wild type (*n* = 36)	*p*
Chemotherapy			
Folfox	16 (80%)	32 (89%)	*p* = 0.61
Folfiri	10 (50%)	19 (53%)	*p* = 0.94
Cetuximab	19 (95%)	30 (83%)	*p* = 0.40
5FU	1 (5%)	3 (8%)	*p* = 0.94
Bevacizumab	8 (40%)	20 (56%)	*p* = 0.40
Surgery			
Primary resection	13	30	*p* = 0.22

Of the 56 patients included in the study, 43 patients (76.8%) underwent resection of their primary tumour, while 13 patients (23.2%) did not. Thirty-two patients presented with de novo metastatic disease. There was no difference in the overall rate of resection between the 2 groups. All 24 patients across both groups, *BRAF*^MUT^ and *BRAF*^WT^ who did not have metastatic disease at initial presentation underwent resection of the primary. Of those who had metastatic disease at initial diagnosis, 16 of 22 patients (73%) with *BRAF*^WT^ underwent surgical resection, while only 3 of 10 (30%) of those with *BRAF*^MUT^ went on to have primary resection of their tumour (*p* = 0.058).

**Fig. 4:**
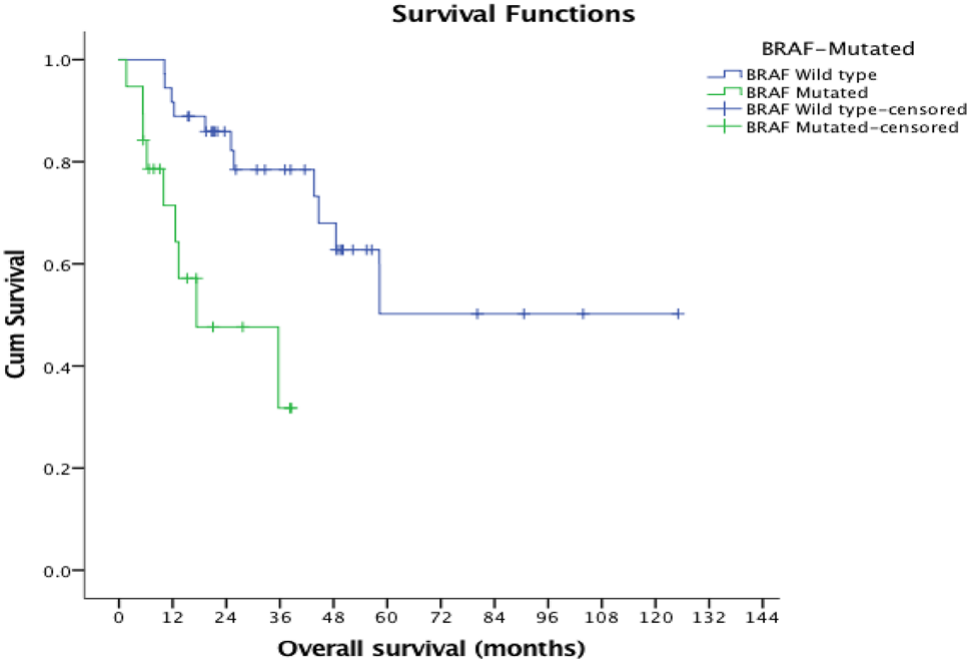
Kaplan Meier Curves showing survival in patients with *BRAF*^MUT^ and *BRAF*^WT^

Figure 4 shows the Kaplan Meier curves for survival in patient with mutated or wild type *BRAF* gene. Significantly poorer outcomes were noted for those with *BRAF*^MUT^ disease, with a median overall survival of 17.3 months (95% CI 0–40.8) when compared with those exhibiting *BRAF*^WT^ disease, median survival not yet reached (log rank *p* = 0.001.).

On multivariate analysis *BRAF*^MUT^ was independently associated with an increased risk of mortality (HR 12.76 (95% CI 3.15–51.7), p < 0.001).

### Appendices

Copies of the data collection sheet and ethical approval form can be found in Appendices 1 and 2 respectively.

## Discussion

This study showed that in an Irish population with advanced CRC, patients in the *BRAF*^MUT^ cohort were predominantly of female gender (p = 0.007). This is an interesting finding, given the incidence rate of CRC is known to be higher in Irish males than females (65.2% vs 39.9%) [2]. A 2014 meta-analysis of 25 international studies by Chen et al. found there was a significant association between *BRAF*^V600E^ and female gender, with the *BRAF*^V600E^ mutation 1.71 fold more frequent in females than males (OR = 1.71; 95% CI = 1.42–2.07) [21]. Furthermore, Tie et al. noted that female gender was an independent predictor of *BRAF*^MUT^ in these patients [22].

*BRAF*^MUT^ tumours in this study were found to be principally right-sided in origin in comparison with the *BRAF*^WT^ group, where the opposite was true (p = 0.033). A 2011 paper by Tie and Desai illustrated that right-sided tumour location was an independent risk prediction of *BRAF*^MUT^ status. Furthermore, when variables were controlled for, the prevalence of *BRAF*^MUT^ in selected females with right-colon tumours was 37% [23]. This is highly significant when noting the accepted prevalence of *BRAF*^MUT^ in CRC is approximately 8–15% [16].

This finding is important as the clinical presentation of the tumour is largely dictated by its anatomical location. Left-sided colon tumours are known to present with altered bowel habit and rectal bleeding, while more proximal right-sided tumours are more likely to present insidiously with iron deficiency anaemia [24]. A 2016 analysis of 200 cases of CRC by Hussain et al. found that right-sided colon tumours were inclined to be more aggressive and associated with poorer prognosis than their left-sided counterparts [25]. This may be due to a variety of reasons, including underlying genetic mutations or potential environmental differences including bacterial populations and exposure to bile salts [24]. Recent data from the FIRE-3 and CALGB/SWOG 80,405 confirms that right-sided tumours do worse when compared with left sided tumours [26].

A significant finding of this study was the high incidence of poorly differentiated tumours in the *BRAF*^MUT^ cohort compared with the *BRAF*^WT^ group. A number of studies have also correlated *BRAF*^MUT^ with poorly differentiated tumours, which are known to confer a poorer prognosis than tumours of the well-differentiated subtype [21, 27]. However, it appears that the poor prognosis associated with *BRAF*^MUT^ remains independent of the degree of differentiation. There were no significant differences noted in the distribution of tumours between stages or the presence of metastasis at initial diagnosis in this study, but once again this should be interpreted with caution because of relatively small numbers.

An interesting clinicopathological finding of this study was while 29 patients (81%) of the *BRAF*^WT^ group tested positive for *KRAS* mutation, none of the *BRAF*^MUT^ group did (p < 0.00001). This corroborates evidence in the literature that *BRAF* and *KRAS* mutations are mutually exclusive [23]. However, it should be noted that *KRAS* could be an independent marker for metastatic disease in *BRAF*^WT^ patients. This was outside the scope of this study, but further research is required in this study area.

Patients in both arms underwent surgical resection of the primary tumour and/or chemotherapy administration. Surgical resection of CRC is standard of care unless contraindicated or deemed unsuitable, while standard first-line chemotherapy for metastatic CRC conventionally involves fluorouracil plus folinic acid in combination with irinotecan (FOLFIRI) or in combination with oxaliplatin (FOLFOX). The addition of anti-vascular endothelial growth factor (Anti-VEGF) antibodies, such as bevacizumab, or anti-epidermal growth factor receptor (Anti-EGFR) antibodies such as cetuximab or panitumumab, is common, and a variety of these agents were administered to patients in this study [28, 29].

In our study, 13 patients in the *BRAF*^MUT^ group had their primary tumour resected compared with 30 patients in the *BRAF*^WT^ group. While this was not significant (p = 0.22), it still poses an interesting finding, raising the question of why fewer *BRAF*^MUT^ patients underwent surgical resection of primaries compared with *BRAF*^WT^ patients in this population. All 24 patients with localised disease at initial presentation had their primary tumours resected. However, when only those with metastatic disease at initial presentation were included, 73% with *BRAF*^WT^ and only 30% of those with *BRAF*^MUT^ went on to have primary resection of their tumours (p = 0.058). The rate of resection of the primary tumour in the *BRAF*^WT^ group is relatively high. Even in incurable stage IV disease, palliative primary tumour resection is associated with improved survival [30] and it is our institutional policy to resect the primary tumour if possible, usually after neoadjuvant chemotherapy. In some cases, the metastatic tumour progressed during neoadjuvant chemotherapy, and resection of the primary was not performed. The lower rate of subsequent primary tumour resection in *BRAF*^MUT^ patients who presented with metastatic disease is most likely related to the more aggressive nature of the disease in these patients. The granularity of the data in this retrospective study is insufficient to draw more detailed conclusions, although this certainly warrants further study.

Table 2 indicates the therapeutic agents used. Whilst there were no significant differences between groups, insufficient data was available to derive meaningful conclusions, and further study is required in relation to this. Nonetheless, a number of exciting clinical trials exist in relation to oncological treatment of *BRAF*^MUT^ CRC. A crucial finding in these trials has been that the presence of a *BRAF* mutation in CRC has been linked with poor response to conventional chemotherapeutic agents. It has also been hypothesised that *BRAF*^MUT^ may be resistant to anti-EGFR agents, though a definitive link has not yet been established [23].

Both the CRYSTAL and OPUS trials examined the effects of adding cetuximab, an EGFR inhibitor, to conventional chemotherapy in CRC. These large-scale studies observed that while adding cetuximab to chemotherapy led to significantly improved outcomes in *BRAF*^WT^ disease in comparison to administering chemotherapy alone, *BRAF*^MUT^ tumours were associated with worse prognosis in both treatment regimes (median overall survival (OS) 9.9/14.1 months respectively, compared with 21.1/24.8 months in *BRAF*^WT^) [31]. Furthermore, the CAIRO-II study examined the addition of cetuximab to triple therapy of Capecitabine, Oxaliplatin, and Bevacizumab, observing reduced OS of 15.2 months (*BRAF*^MUT^) compared with 21.5 months (*BRAF*^WT^) in the cetuximab group. *BRAF*^MUT^ was also discovered to confer a poor prognosis independently of cetuximab [23, 32, 33]. These trials certainly provide evidence for the poor outcomes observed in our study; however, detailed analysis outside the scope of this study would be required to compare results.

While the issue of relative chemo-insensitivity among this specific patient population represents a considerable obstacle at the forefront of oncological research, promising new data has been reported. The recent BEACON trial hypothesised that utilising triple-therapy with three chemotherapeutic agents; encorafenib, binimetinib, and cetuximab in patients with *BRAF*^MUT^ CRC, who had progression of disease following initial chemotherapy regimens, increased overall survival. The large-scale study comprising 665 patients found the median-overall survival to be 9 months in the triplet group compared with 5.4 months in the control group (hazard ratio for death, 0.52; 95% CI 0.39–0.70: p < 0.001). It was also discovered that the response rate to these agents was considerably higher at 26% (95% CI 18–35) than in the group treated with one agent, whose response stood at 2% (95% CI 0–7) [16]. A recent update on the BEACON trial showed that the doublet regimen of encorafenib and cetuximab had similar efficacy to the triplet regimen including binimetinib. The doublet regimen is now accepted as the optimal regimen in this patient population and was FDA approved in 2020 [34]. *BRAF*^MUT^ in colorectal cancer correlates with reduced overall survival in comparison to CRC patients without the mutation [8–10]. This was also a key finding of our study. In the previously discussed study by Atreya et al., mean OS was noted to be 24 months in the *BRAF*^MUT^ group when compared with 45 months in the *BRAF*^WT^ group [18]. Previously, a similar study by Souglakos et al. noted median (OS) in their *BRAF*^MUT^ group to be 14 months compared with 30 months in their *BRAF*^WT^ control. A further finding by this study was that on multivariate analysis, *BRAF*^MUT^ was found to be an independent negative prognostic factor for overall survival [19]. This finding was replicated in our study.

Triplet agent FOLFOXIRI was not given to any of the patients involved in this study as it was felt the toxicity from such a regimen would outweigh the benefits derived from it. A recent meta-analysis comparing a triplet regime of FOLFOXIRI plus bevacizumab to a doublet regime plus bevacizumab failed to show any benefit of the triplet regime over the doublet regime [35]. A recent review of treatment options and evidence-based guidelines by Grothey et al. noted that current NCCN guidelines recommend combination chemotherapy as first-line in the treatment of patients with metastatic colorectal cancer, including those harbouring the *BRAF* mutation [36]. Triplet regimens containing dabrafenib plus trametinib plus cetuximab, or panitumumab and encorafenib plus binimetinib plus cetuximab or panitumumab were removed as treatment options for *BRAF*-mutated CRC, based on recommendations resulting from the recent BEACON CRC trial [34].

A large-scale study of 524 mCRC patients by Tran et al. noted that there was a high concordance of *BRAF*^MUT^ CRC with peritoneal metastasis and involvement of distal lymph nodes, but low levels of lung involvement [37]. It is widely known that involvement of the peritoneum in metastatic CRC is a poor prognostic indicator for survival, but whether *BRAF*^MUT^ is an independent risk factor for peritoneal involvement remains to be seen [38]. The high concordance between the two factors may somewhat explain the inferior outcomes in survival in this patient group, and may be of interest for further study. An interesting study by Yaeger et al. questioned the role of liver resection for *BRAF* mutated CRC which also provides scope for further study [8].

Identification of the specific subtype of Irish CRC patients likely to develop a *BRAF* mutation was a fundamental finding of this report, this being females over the age of 50 presenting with right-sided tumours. This information is beneficial in order both to expand the knowledge basis and to provide the ability to optimise clinical management of Irish CRC patients. Both the findings of this report and international findings agree that these tumours will metastasise early, respond sub-optimally to surgical and oncological treatment, and likely have shorter survivals [8–10]. Therefore, current clinical practice must be adjusted in order to take this into account.

Given the relative insensitivity of *BRAF* CRC to conventional chemotherapeutic agents, one could hypothesis that the early introduction of a targeted therapeutic strategy may be more effective. Thus far, the majority of research conducted has focused on the implication of *BRAF*^MUT^ in late or metastatic disease. An area of research in which there remains a paucity of data is the implications of harbouring a *BRAF*^MUT^ in early-stage disease. Perhaps the question of whether to routinely screen all CRC patients in high-risk subgroups for the *BRAF* mutation, regardless of stage, should also be asked.

Strengths of this study included it being a multi-centre study, with patients undergoing treatment at both CUH and MUH included in the population. Standard protocols were used throughout data collection and analysis, and a multivariate analysis was utilised to control for variables. Despite the many strengths, this study was also limited by a number of factors. The retrospective nature of the data collection did not allow for confounding factors to be controlled for. This was further limited by the small sample size available due to the low prevalence of the mutation. Nevertheless, this study presents some important and significant findings.

In conclusion, in accordance with the international literature, *BRAF*^MUT^ conferred a poorer prognosis than *BRAF*^WT^ in this Irish population. *BRAF*^MUT^ tumours were primarily right sided in origin, had a higher incidence in females and had a higher incidence of poorly differentiated tumours when compared with the *BRAF*^WT^ group. While the *BRAF*^MUT^ rate in our study population was low, *BRAF*^MUT^ colorectal cancer has been shown to be an aggressive form of cancer, despite the small numbers involved. Knowledge of *BRAF* status should be standard of care in all metastatic colorectal cancer patients as it is now known to be an adverse prognostic factor, and may play a future role in the management of early stage disease. *BRAF* testing should therefore be conducted routinely in Irish metastatic colorectal cancer patients, as it has the potential to guide therapeutic strategy in this small but important group of patients. Promising new targeted therapies have recently been approved and will most likely become standard of care.

## Data Availability

Data required for this study was sought from the pathology database at Cork University Hospital. This was made available on receipt of ethical approval, granted by the Cork Research Ethics Committee (CREC). More Information available in the report.

## Code availability

Statistical analysis was conducted using SPSS version 25 (SPSS, Inc., Chicago, Illinois).
